# Severe sepsis with *Hafnia alvei* and dual pulmonary infection with *Mycobacterium tuberculosis* and *Pneumocystis carinii* in a late presenter patient infected with HIV – case presentation

**DOI:** 10.1186/1471-2334-14-S4-P21

**Published:** 2014-05-29

**Authors:** Andreea Ardeleanu, Ovidiu Roşca, Monica Cialma, Daniela Desagă, Ingrid Deak

**Affiliations:** 1 Department of Infectious Diseases I, Dr. Victor Babeş Clinical Hospital of Infectious Diseases and Pneumology, Timişoara, Romania; 2 Department of Infectious Diseases I, Victor Babeş University of Medicine and Pharmacy, Timişoara, Romania; 3 City Hospital Vulcan, Hunedoara, Romania

## 

Once thought to be a simple commensal of the gastrointestinal tract, there is increasing evidence to suggest *Hafnia alvei* is a rare, but significant bacterium that may contribute to opportunistic infections in humans. Bacteremia and respiratory tract infections are the leading extraintestinal manifestations of *Hafnia alvei* as a pathogen. It can cause disease in immunocompromised patients and it is regularly resistant to multiple drugs.

Patients with HIV may present with both TB and PCP and in these patients, TB seems to account for the most serious symptoms of their disease that require hospitalization.

We report the case of a 36-years old patient admitted in our department in 23 October 2013, for persistent fever, headache, oral candidiasis, productive cough, weight loss and severe asthenia. He tested positive for HIV with a CD4 of 7 cells/µL and a viral load of 930,795 copies/mL. In the blood culture we isolated *Hafnia alvei* and the chest CT scan showed “a ground-glass aspect” suggestive for a *Pneumocystis carinii* pneumonia (PCP). The pneumological evaluation established the imagistic diagnostic of miliary tuberculosis and recommended treatment with tuberculostatic drugs and antibiotic.

Severe sepsis has emerged as a common cause of hospital admission for those living with HIV/AIDS. Sepsis patients had significantly higher in-hospital mortality than did nonsepsis patients. Clinicians should be aware that patients with HIV/AIDS may present with concurrent pulmonary TB and PCP, especially in regions that are hyperendemic for TB.

